# Paediatric family activation rapid response (FARR) in tertiary healthcare organisations: Protocol for an online, multi-lingual, application (app) intervention development study

**DOI:** 10.1186/s12887-023-04123-w

**Published:** 2023-06-17

**Authors:** Takawira C. Marufu, Nicola Taylor, Shannon Cresham Fox, Rachel Boardman, Joseph C. Manning

**Affiliations:** 1 Nottingham Children’s Hospital, Nottingham University Hospitals NHS Trust, Room SC3084, C-Floor, South Block, Queen’s Medical Centre Campus, Derby Road, Nottingham, NG7 2UH UK; 2 Children and Young People Health Research, School of Health Sciences, Faculty of Medicine and Health Sciences, The University of Nottingham, Nottingham, UK

**Keywords:** Family activation, Rapid response, Children, Pediatrics

## Abstract

**Introduction:**

At least 85% of unplanned admissions to critical care wards for children and young people (CYP) are associated with clinical deterioration. CYP and their families play an integral role in the recognition of deterioration. The Paediatric Critical Care Outreach Team (PCCOT) supports the reduction of avoidable harm through earlier recognition and treatment of the deteriorating child, acting as a welcome conduit between the multiprofessional teams, helping ensure that CYP gets the right care, at the right time and in the right place. This positions PCCOT well to respond to families who call for help as part of family activation.

**Aim:**

This protocol details the methods and process of developing a family activation rapid response online application.

**Methods:**

This is a single-centre, sequential, multiple methods study design. Firstly, a systematic review of the international literature on rapid response interventions in paediatric family activation was conducted. Findings from the review aimed to inform the content for next stages; interviews/ focus groups and experience-based co-design (EBCD) workshops. Participants: parents / caregivers whose children have been discharged or admitted to an acute care hospital and healthcare professionals who care for paediatric patients (CYP). During interviews and workshops participants’ opinion, views and input will be sort on designing a family activation rapid response online-app, detailing content, aesthetics, broad functionality and multi-lingual aspects. Further areas of discussions include; who will use the app, access, appropriate language and terminology for use.

A suitable app development company will be identified and will be part of the stakeholders present at workshops. Data obtained will be used to develop a multi-lingual paediatric family activation rapid response web based application prototype.

**Ethics and dissemination:**

Full ethical approval was received from the Wales Research Ethics Committee 2.

Cardiff; REC reference: 22/WA/0174. The findings will be made available to all stakeholders.

## Introduction

Hospitalised patients may develop potentially avoidable medical and physiological complications that result in unplanned admissions to intensive care, cardiac arrest, and / or sometimes death [1, 2]. Globally this affects approximately 70% of hospital admissions and can potentially be avoidable if signs of clinical deterioration are recognised early, escalated and followed by appropriate and timely treatment (interventions) [2]. This has led to a constellation of complex interventions contributing to a systems approach to recognition and rescue. Implementation of international and national tracking and trigger systems, such as early warning scores (national early warning scores (NEWS) for adult patients [3] and paediatric early warning scores (PEWS) for children and young people (CYP) [4]), and timely activation of appropriately trained staff, such as rapid response team (RRT) mechanisms, have been priority initiatives to improve patient safety [5].

The United Kingdom (UK) Paediatric Intensive Care Audit Network (PICANet) reported that more than 85% of unplanned admissions to paediatric critical care units (PCCU) are commonly associated with acute clinical deterioration [6, 7]. Early detection in the development of a critical illness can reduce the need for critical care as it can lessen the complexities of treatments and improves outcome [5]. While deterioration can often be predicted by regular monitoring of physiological changes, these may not always be recognised or appropriately acted upon [1]. The inability or *failure* of a healthcare organisation or its professionals to identify and recognise early signs of deterioration is described as ‘failure to rescue’, which has been identified and acknowledged as one of the leading causes of death or harm in patients [5, 7, 8].

Consequently, there has been an increasing emphasis on the need for the development or provision of access to mechanisms that allow families (parents and/ or carers) of hospitalised CYP to escalate concerns to reduce adverse events [1]. These initiatives include families mobilising the paediatric RRT of a healthcare organisation, a process known as *the 'family activation rapid response’*. Contemporary literature supports the notion that families often recognise changes in their child’s condition before healthcare staff [5, 9, 10]. It is intuitive to think that families know when *‘something is not quite right’* and can sense if their child’s clinical condition is deteriorating [11]. This is especially true for CYP with complex health needs that have altered normal physiology, where subtle changes are hard for the healthcare worker to detect.

Various family activation rapid response interventions (condition help program [12], calling for help [13], medical emergency (rapid response) teams [14, 15]) [5] have empowered families to help prevent medical errors and breakdown of communication by activating a system that allows them to ‘call for help’ if they do not feel listened to or are concerned about the patient’s condition. The Josie King foundation [16] describes this as developing a 'safety culture together', uniting the healthcare worker and their consumer. However, all these interventions are only telephone-activated and do not consider other languages, even though healthcare institutes cater for multilingual communities.

In view of the important role families can play in contributing to the early recognition of their child’s clinical deterioration, this study aims to develop an innovative web based multi-lingual application (app) mechanism by which families (carers) can escalate their concerns directly to the RRT (Paediatric Critical Care Outreach Team (PCCOT)). This new mechanism will allow families to send messages to the RRT in comparison to telephone use, where sometimes it takes a while for someone to respond. The objective is to enable a rapid response to their concerns and initiate timely rescue interventions as required [17]. The aim is not to bypass or stifle normal communication pathways, but to add a safety net for those times when communication has broken down and family feel that their concerns have not been heard.

For the purpose of this study, family activation (FA) of a rapid response system is described as escalation of deterioration concerns of a hospitalised child directly to PCCOT by a family member / caregiver via an online web or mobile app-based system.

### Study aim and objectives

#### Aim

To develop a multilingual family activation (FA) rapid response system, to facilitate early recognition and rescue of the deteriorating child through family participation in raising concerns to PCCOT through an app-based system.

#### Objectives

(i) Conduct a comprehensive systematic review of the current international literature on family activation rapid response interventions
(ii) To identify and recruit participants that meet predefined study eligibility criteria.
(iii) To conduct interviews with participants on their views and perceptions on overall app appearance, look and functionality.
(iv) To conduct experience-based co-design (EBCD) workshops with participants for their views and perceptions on app development
(v) To ensure inclusion of a language translation option in the app framework, to give families a choice of the language to use

### Theoretical framework

Family activation rapid response is a complex intervention due to various reasons which include; expertise and skills required by those delivering and receiving the intervention; settings and the number of components involved. As such, this study used the Medical Research Council (MRC) framework for the development and evaluation of complex interventions [18] as guidance on the planning and delivery of the study. According to the framework, this study is in the intervention development stage, with some focus on identifying the evidence base. The following framework core elements; context, stakeholders engagement and development and refinement of the intervention are clearly outlined in study planning and delivery.

## Methods

### Study setting

The study setting is a 110-bed children's hospital that offers a range of services for CYP of age ranging from 0 to 18 years and is part of an acute UK hospital (including a trauma centre). The hospital admits approximately 22 000 paediatric inpatients per year. This is a single centre co-design app development study. All study activities will be conducted and co-ordinated within the children’s hospital.

To achieve the desired outcome this project will use an Experience-Based Co-Design (EBCD) approach, which aims to combine a user-centred orientation (Experience Based (EB)), and a collaborative change process (Co-Design (CD)) [19, 20]. All stakeholders have a participatory role working together in designing the FA app (co-design rather than re-design). The following stages as part of the design process will be followed; project setup, evidence gathering, bringing parents / caregivers and staff together to share experiences and begin co-design, coming back together for celebration and review [19]. The study conduct complied with the standards of Good Clinical Practice (GCP) set forth in the Declaration of Helsinki of 1975 [21].

### Participant recruitment

Parents / caregivers and healthcare professionals (including digital experts) will participate in this study. Table 1 below details full participants eligibility criteria.

1. Parents / caregivers whose children have been discharged or are being discharged from acute inpatient care and those whose children are still inpatients are eligible for inclusion in the study. Parents/ and or carer participants will be identified by the usual care team during outpatients or clinic visits, on the day their child is being discharged from the hospital or approached whilst they have a child who is an inpatient. For hospitalised children known to PCCOT, their parents will be identified by PCCOT. Once a potential candidate is identified, their usual care team will contact them, who will inform them of the study.

**Table 1:** Participant eligibility criteria

Study population	Inclusion criteria	Exclusion criteria
Parents(families) /career	• Parents with hospitalised children known to the paediatric critical care outreach team (PCCOT)/ Parents with hospitalised children who have had a referral or review by PCCOT • Parents (families)/ carers of children discharged from hospital • Parents (families) who currently have children or young people who are inpatients • Ability to give informed consent prior to enrolment in the study	• Not able to give informed consent • Parents of children who are not inpatients or have not been admitted to hospital before
Healthcare professionals	• Healthcare professionals caring for hospitalised children • IT professionals working in the hospital • Ability to give informed consent before study enrolment	• Unable to give informed consent • Healthcare professionals not looking after hospitalised children OR not IT staff

Patient information sheet (relatives/ carers participant information) will be given with the focus group sessions schedule and information about individual interviews. All potential participants will have time to consider whether they want to participate in the study. For those who leave the hospital without making the decision, the usual care team will seek the permission of those potential participants to follow them up with a phone call using their currently held contact details to enquire if they are willing to participate in the study. If potential participants are willing to take part, the member of the usual care team who is part of the research team will obtain verbal consent to enrol the participant into the study. Parents of children known to PCCOT who are enrolled into the study will be given an option to be interviewed while their child is still in hospital or on the day of discharge.

Participants will also be recruited via the hospital patient public involvement and engagement (PPIE) groups. Study information will be sent to the PPIE groups and patient partnership groups through the hospital PPIE lead. Once the information is made available, those who are eligible and willing to participate will contact the research team for further information or to give consent and enrol in the study.

The PPIE groups includes the hospitals’ Black, Asian and minority ethnic shared governance council who have contact with various community groups. Study information with eligibility criteria and workshop schedule will be made available and participants willing to take part in the study will be able to contact the study team via email, telephone or text, to give consent and enrol in the study.

To support and inform the multilingual aspect of product development, study participants information sheet and study posters will be translated into Urdu and Polish languages, which are app language options. These two languages were chosen in reflection to the local community representation.

2. Healthcare professionals who care for paediatric patients (children and young people (CYP)) and information technology (IT) professionals who work in the hospital will be recruited for the study. The clinical departments will be visited. A staff information sheet will be provided, and posters with study information and upcoming scheduled focus group sessions will be displayed in the staff rooms and near the main entrance.

### Eligibility criteria

#### Sample size

The study aims for a maximum sample size of 30 participants (estimated at 10 healthcare professionals and 20 parents/ carers). If required, workshops will be separated targeting individual stakeholder groups. This is for consideration in cases where parents may not feel confident to say what they want if healthcare staff are present. Participants will be purposively sampled [22] using the eligibility criteria outlined.

Where possible, the healthcare professional participant group will be stratified according to clinical specialty and staff group (doctors, nurses, IT). This will be achieved by ensuring that at least three different clinical departments are represented and by recruiting a similar number of participants from each department where applicable.

#### Informed consent

All participants will provide their informed consent prior to participating in the study. If possible, written informed consent will be provided. The consent form will be signed and dated by the participant before entering the study. Alternatively verbal consent from potential participants will be obtained over the telephone, especially in-case of parents/carers whose children have been discharged. If potential participants speak another language we will utilise the local hospital language translation services to support involvement and information sharing.

The investigator will explain the details of the study and provide a participant information sheet, ensuring that the participant has sufficient time to consider participating or not. The investigator will answer any questions the participant has about participation in the study.

When written informed consent is provided, a copy will be kept by the participant and a copy will be kept by the investigator. Where it is not possible to obtain written consent, verbal consent will be obtained. Verbal instruction and contact details will be received from participants who have received the participant information sheet through PPIE networks and want to participate in the study. If they have any questions or need clarification, this will be done before verbal consent is obtained by telephone.

For verbal consent obtained by telephone, a copy of the completed consent form will be sent to the participant by post.

Should there be any subsequent amendment to the final protocol, which might affect a participant’s participation in the study, continuing consent will be obtained using an amended consent form which will be signed or agreed over the telephone by the participants.

### Data collection

#### Systematic review of contemporary evidence

A comprehensive systematic review of the current international literature on family activation rapid response interventions has been carried out and recently published [5]. The review protocol has been registered with PROSPERO; registration number: CRD42022304695. Review findings will be used to inform focus groups, interviews and ECBD workshop discussions to effect app development.

#### Focus groups and interviews

As part of the exploratory phase [18], focus groups / individual interviews will be conducted with all stakeholders to gain an understanding of what family activation of rapid response means to them. Focus groups/ interviews were deemed appropriate to capture nuanced and contextual perceptions of all stakeholders [17].

The aim of the focus groups/ interviews is to build content and remit of the intervention; that is content and stakeholders’ expectation on designing the rapid response FA app, its appearance and how it works. This will also include who will use the app, how it will be accessed, appropriate language and wording for use, how to reduce any potential barriers, the process and mechanism to ensure timely communication is maintained, and potential safety concerns. Details on how the rapid response FA app will be helpful, as a new system will also be explored**.** Individual interviews and focus groups will last 15 to 30 min and 30 to 60 min respectively.

#### EBCD workshops

Three progressive co-design workshops lasting 30 to 60 min will be conducted. The primary purpose of the workshops will be to design and develop the intervention. Where required further workshop sessions will be conducted to facilitate contribution of participants who may have missed the three workshops.

Interviews, focus groups and workshops will be delivered face to face, over the phone or virtually (via Cisco Webex) or any other appropriate secure platform approved for use by hospital information governance (IG). Participants will also be able to switch off their cameras during online focus group discussions/ interviews.

Green and Thorogood [23] stated that the experience of most qualitative researchers conducting focus group based interview studies is that little new information is generated after interviewing 20 participants or so belonging to one analytical relevant participant group. We aim to have an average of 5 – 15 participants per focus group session. Having a maximum number of 15 participants per session within the allocated workshop timeframe will enable all participants ample time to fully express their views [24]. Focus groups will be moderated by the chief investigator, co-investigators or project research assistance. Online focus group and virtual interview conversations will be video and audio-recorded and field notes will be collected. Face to face and telephone interviews will be audio recoded using a Trust IG approved recorder. All information collected will be kept confidential. The recordings will be kept on a hospital network accessed by the study team only using a password and the written documents will be kept in the study office in a locked cupboard.

Investigators will follow ethical and legal practice and all information about participants will be handled in confidence. All identifiable participant information will be removed at the data transcription stage. All interviews transcripts for analysis will use unique participant codes to ensure that participants cannot be recognised from it.

### Data analysis

The characteristics of the participants (demographics) and data from the reports will be reported as numbers (n) and percentages (%), including the mean, standard deviation (SD), and range where applicable. Data recorded from interviews, focus groups and workshops will be analysed through content coding by the research team to develop themes from participant groups drawing on a grounded theory approach [25]. The investigators will perform content analysis to develop themes, trends, and patterns on; the environmental context, resources, mechanism, process, participant experiences and communication, their frequency and their relationships will be used for analysis [26]. Furthermore, observed themes will be cross-checked using the following framework on good EBCD experiences of new tools [20];

(i) Performance (P) – how well it does the job/ is it for purpose (functionality and efficiency)
(ii) Engineering (E) – how safe, well-engineered and reliable it is (safety), interface
(iii) The aesthetics of experience (A) – how the whole interaction with the product/ service feels/ is experienced (physical environment and human environment), (experience).

### FA theoretical conceptual framework

Empirical-led family activation themes obtained from data analysis will be compared and contrasted and combined with literature-led themes to develop a new overarching paediatric FARR conceptual framework.

### Ethics

All study documents (protocol, participant information sheets, consent forms, posters) have receive full NHS Research Ethics Committee (REC) approval from the Wales Research Ethics Committee 2 Cardiff; (REC reference: 22/WA/0174), and local approval from Nottingham University Hospitals Research and Innovation department before study commencement. Substantial amendments that require review by REC will not be implemented until the REC grants a favourable opinion. All correspondence with the REC is retained.

An annual progress report (APR) will be submitted to the REC within 30 days of the anniversary date on which the favourable opinion was given, and annually until the study is declared to be ended. It is the responsibility of the chief investigator to produce annual reports as required. The Chief Investigator will notify the REC of the end of the study. If the study is ended prematurely, the Chief Investigator will notify the REC, including the reasons for the premature termination. Within one year after the end of the study, the chief investigator will submit a final report with the results, including any publications/abstracts, to the REC.

The study will be monitored and audited by the sponsor as required.

#### Assessment and management of risk

As part of the workshop and focus group discussions, the study team recognise that for some of the parents and or carers, their experiences maybe distressing, as they might relive their experiences of when their child was in hospital. To mitigate the risk; the participant information sheets from relatives / caregivers clearly state that discussing experiences after their child's hospitalisation can be distressing and we will ask participants to carefully consider how they feel about this prospect before deciding to participate. Study workshops will be facilitated by experienced paediatric nursing staff who also have experience in supporting families and caregivers during such circumstances and any training required in the use of specific methodologies will be provided to any other researchers in this study [27]. Researchers will be particularly sensitive to and careful about how these experiences are explored. If the participants wanted someone else to be present with them, this would be facilitated. Sessions will be facilitated in a form that will allow for the building of relationships with participants at the beginning and appropriate end of the session before leaving. Any further support required participants will be signposted and or advised to seek help from their General Practitioner (GP).

The overall aim of the study is to develop a multilingual FA app. To allow inclusion of those who may not adequately understand verbal explanations or written information in English, the study will use hospital translation services to support inclusion. Where applicable, we will also consider using family members to offer support to potential participants to bring clarity to the conversation. If participants would like someone else to be present with them during the consent process or focus group/ interview sessions, this would be facilitated. Verbal consent will be obtained from these family members.

### Data protection and confidentiality

All study staff and investigators will strive to protect the rights of study participants to privacy and informed consent and will adhere to the General Data Protection Regulation 2018 and the Data Protection Act 2018. All virtual focus groups/ interviews will be recorded online using hospital Trust IG approved methods, and the case report form (CRF) will only collect the minimum required information (demographic data) for the purposes of the study. The CRFs will be securely held in a locked room, or a locked cabinet or cabinet. Access to the information will be limited to the study staff and investigators and any relevant regulatory authorities. Computer-held data, including workshop audios, will be kept securely and password protected. All data will be stored on the hospital secure web server. Access will be restricted by user identifiers and passwords (encrypted using a one way encryption method).

Data will be stored and retained for five years in compliance with the ICH/GCP guidance and will be archived as per hospital policy. Arrangements for confidential destruction will be made after 5 years.

### Patient and public involvement

This project uses the co-design methodology that underpins Patient and Public Involvement and Engagement (PPIE) at every stage of FA app development. PPIE is a central part of this project with strategic direction provided by the lead teams of several PPIE groups in the hospital that will be involved in the project (IT PPI group, hospital PPI group) and the BAME shared governance council.

The Nottingham University Hospital as an organisation has established a culture of meaningful PPIE that employ Experience Based Co-Design methodology [12]. This study has been designed with the participation of stakeholders (service users’) involvement in mind to provide direction on the research that is being carried out for the study to achieve an FA app relevant and best suited for their use. We are committed to ensuring that a diverse population of parents and carers is involved in this project specifically targeting underserved communities in line with hospital Equality, Diversity, and Inclusivity Strategy.

### Dissemination

Preliminary reports will be presented at relevant national or international meetings. Full scientific papers will be published in peer-reviewed journals. Participants will not be identified in any publications. Participants will be given research team details and we will supply them with research summaries if and when requested. It is anticipated that preliminary reports will be published within 6 months of study completion; full papers are expected to take up to 12–18 months for publication. The prototype version of the family activation rapid response app developed will be shared with the sponsors and national and international partners.

## Data Availability

Not applicable.
